# Increased PD‐1/PD‐L1 Immune Checkpoint Expression Is Associated With Oral Squamous Cell Carcinoma in Never‐Smokers and Never‐Drinkers

**DOI:** 10.1002/hed.27981

**Published:** 2024-10-27

**Authors:** Mathias Fiedler, Alisa Off, Andreas Gärtner, Gero Brockhoff, Jonas Eichberger, Maximilian Gottsauner, Johannes G. Schuderer, Michael Maurer, Richard J. Bauer, Michael Gerken, Torsten E. Reichert, Tobias Ettl, Florian Weber

**Affiliations:** ^1^ Department of Oral and Maxillofacial Surgery University Hospital Regensburg Regensburg Germany; ^2^ Institute of Pathology University of Regensburg Regensburg Germany; ^3^ Clinic of Gynecology and Obstetrics, Caritas Hospital St. Josef University of Regensburg Regensburg Germany; ^4^ Department of Oral and Maxillofacial Surgery, Center for Medical Biotechnology University Hospital Regensburg Regensburg Germany; ^5^ Center of Tumor Registry University of Regensburg Regensburg Germany

**Keywords:** never‐drinkers, never‐smoker, Oral squamous cell carcinoma, PD‐1, PD‐L1

## Abstract

**Background:**

This study aimed to explore the disparities in PD‐1 and PD‐L1 expression among oral squamous cell carcinomas (OSCCs) in individuals categorized as never‐smokers/never‐drinkers versus smokers/drinkers.

**Methods:**

Immunohistochemical staining for PD‐1 and PD‐L1, along with PDCD1LG2/cen9 dual color probe analysis, was conducted on 130 OSCC specimens from both smoker/drinker and never‐smoker/never‐drinker cohorts. Associations between smoking/drinking status, clinicopathologic data, immunohistochemical antibody expression, fluorescence in situ hybridization, and survival outcomes were assessed.

**Results:**

OSCC in never‐smokers/never‐drinkers exhibited significantly elevated PD‐1 expression (*p* = 0.003), increased PD‐L1‐TPS expression (*p* = 0.044), and elevated PD‐L1‐CPS expression (*p* < 0.001). High PD‐L1‐ICS expression was more prevalent in never‐smokers (*p* = 0.042). Moreover, never‐smokers and never‐drinkers demonstrated augmented PD‐L1 gene copy numbers (*p* = 0.081 and *p* = 0.054, respectively). Increased PD‐L1 gene copy number, particularly amplification, correlated with PD‐L1‐TPS (*p* = 0.039 and *p* < 0.001). Conversely, PD‐L1 gene copy loss was associated with negative PD‐L1‐CPS (*p* = 0.023). Notably, positive PD‐L1‐CPS was significantly linked with improved overall survival (*p* = 0.023).

**Conclusions:**

OSCC arising in never‐smokers/never‐drinkers exhibit heightened PD‐1/PD‐L1 signaling, suggesting potential efficacy of immune checkpoint therapy in this subgroup of tumors.

## 1 Introduction

A history of alcohol and tobacco use is typical in patients with oral squamous cell carcinoma (OSCC) [1]. Tobacco use is a major cause of cancer development by inducing driver mutations in oncogenes [2]. Ethanol has a carcinogenic effect due to the acetaldehyde produced during its breakdown [3]. Because ethanol acts as a solvent for the carcinogens in tobacco, the risk of developing OSCC can increase up to 35‐fold when both alcohol and nicotine are abused [3, 4]. However, 10%–15% of all patients with OSCC have never smoked or consumed alcohol above the recommended limits (never‐smokers‐never‐drinkers = NSND) [5]. These patients have distinct clinical and histopathological parameters [5, 6]. Among others, NSND patients are increasingly represented in age cohorts that are considered “younger” and “older” when compared to conventional smoker/drinker patients (SD). Additionally, there has been an increase in the number of female patients [7, 8, 9]. Chronic inflammation in the past seems to contribute to carcinogenesis, as NSND‐OSCC patients are more prone to oral potentially malignant disorders (OPMDs) [10, 11, 12, 13]. There are also histopathological differences between “atypical” NSND‐OSCCs and “typical” noxae‐associated carcinomas. According to recent studies, lymphocytes such as CD4‐positive T‐helper cells, CD8‐positive cytotoxic T‐cells, and FoxP3‐positive regulatory T‐cells are increasingly infiltrating the NSND‐OSCC [5, 6]. Another previous finding is that interferon‐*γ* pathway genes are upregulated in NSND‐OSCC, suggesting a greater role for the Programmed Death‐1/Programmed Death‐1 Ligand‐1 (PD‐1/PD‐L1) immune‐checkpoint [5]. In recent years the treatment of OSCC has seen remarkable therapeutic successes with the discovery of immune‐checkpoints and the introduction of PD‐1/PD‐L1 antibodies. However, the response rate remains at about 20% [14]. In clinical practice, the decision to use immunotherapy is often based on the combined‐positive score (CPS) or tumor‐proportion score (TPS) of PD‐L1 [15, 16]. Clinical trials, such as KEYNOTE‐048, have shown that the presence of PD‐L1 (with a cut‐off value of ≥ 1%) on both tumor and tumor‐infiltrating immune cells is associated with a better response to therapy [15, 16]. Therefore, patients with NSND‐OSCC could be a group that may benefit from checkpoint therapy. A precise understanding of the role of the PD‐1/PD‐L1 immune‐checkpoint in NSND‐OSCC could significantly enhance the therapy and prognosis of this patient group. The objective of this study was to examine the expression of PD‐1 and PD‐L1 in patients with OSCC and identify differences between NSND and SD patients.

## 2 Material and Methods

### 2.1 Patient Data Collection

The cohort for the study comprised of 130 patients with OSCC of varying localizations. These patients were diagnosed and staged between 2008 and 2021 at the Department of Oral and Maxillofacial Surgery, University Hospital Regensburg (Regensburg, Germany). Clinical and histopathologic data were collected from the patients' medical records, while follow‐up data were obtained from the Regensburg Tumor Registry. The study received approval from the local ethics committee (No. 22–2798‐101). Never‐smokers are patients who have never used any form of tobacco. Never‐drinkers are individuals who consume no more than 20 g of ethanol per day on average if they are men, or 10 g per day if they are women. The study adhered to the ethical standards of the Helsinki Declaration of 1964 and its subsequent amendments.

### 2.2 Immunohistochemistry

For all patients, formalin‐fixed, paraffin‐embedded tumor tissue was available. The respective samples were obtained from the archive of the Institute of Pathology at the University of Regensburg and tissue microarrays (TMA) were prepared according to the standard protocol as previously described [6, 17]. The tumor region was selected by an experienced pathologist (W.F.) for the preparation of the TMAs.

Slides from each TMA were subjected to immunohistochemical staining with a BenchMark Ultra IHC/ISH system (Ventana Medical Systems, Inc., Tucson, AZ) using the standard protocol of the Institute of Pathology, University of Regensburg [6]. Following antibodies were incubated: PD‐L1 (Dako monoclonal mouse anti‐human PD‐L1 Clone 22C3, Dako North America, Inc., CA, USA) diluted 1:50; PD‐1 (abcam anti‐PD‐1 monoclonal mouse antibody (NAT105) ab52587, Abcam plc, UK) diluted 1:100.

PD‐L1 and PD‐1 were evaluated separately by an expert pathologist and a trained medical professional. Only intra‐ and peritumoral lymphocytes were evaluated. The stroma was not included in the analysis. The observers were blinded, and any controversial cases were discussed. Each patient's three tissue punches were evaluated separately for each antibody, and the average of the three punches was calculated. The evaluation of PD‐L1 was performed over the entire sample. PD‐L1 staining on immune cells and epithelial cells was linear and membranous. Cytoplasmic staining was not considered positive. The intensity of the staining was not considered in the scoring. For PD‐L1 the TPS, the Immune Cell Score (ICS) and the CPS were determined:

‐ TPS (%) = PD‐L1‐positive tumor cells/total number of vital tumor cells × 100.
‐ ICS (%) = area of PD‐L1‐positive immune cells/total area of vital tumor cells × 100.
‐ CPS = (PD‐L1‐positive tumor cells + PD‐L1‐positive immune cells)/total tumor cells × 100.

For statistical analyses, a dichotomization was performed. The TPS was considered as high when the value was ≥ 50%. The ICS was classified as high if the value was ≥ 1%. A high CPS was assumed when the value was ≥ 1. For PD‐1 evaluation, the absolute number of intratumoral cells that were stained on the membrane was manually counted in a high‐power field (HPF) at 400 × magnification. The HPF represents the area of the tumor where most of the cells of interest are located. This area was chosen to achieve the highest possible reproducibility. The evaluation area at 400 × magnification corresponded to a rectangle with a height of 256 μm and a width of 432 μm for the microscope used. A dichotomization into low and high expression groups was performed based on the median value of all tumor samples (low expression ≤ median), which was 21.1667. Immunostaining for CD4, CD8, FoxP3, CD1a, and p16 was performed and evaluated previously [6].

### 2.3 Fluorescence In Situ Hybridization (FISH)

FISH was performed following the protocol as previously described (Table 1) [18]. The PDCD1LG2/cen9 dual color probe (ZytoVision GmbH, Bremerhaven, Germany) labeled with SpectrumGreen and SpectrumOrange for PDCD1LG2 and cen9, respectively, was used for FISH. The number of PD‐L1 gene copies can be accurately determined by the PDCD1LG2 hybridization spots, while the cen9 spots provide a reliable indication of the number of chromosome 9 within a cell nucleus.

**TABLE 1:** FISH protocol.

Step	Conditions	Duration
Deparaffinization	—	—
Pretreatment	0.01 N Na‐Citrate buffer at 98°C	30 min
Incubation with pepsin	Pepsin (ZytoVision Ltd., Bremerhaven, Germany) at 37°C	5 min
Washing	Millipore water	—
Dehydration	Ethanol (70%, 80%, and 100%)	—
Addition of probe	10 μL of the original probe	—
Slide covering	Cover glass and fixogum rubber cement	—
Denaturation	73°C	5 min
Incubation	37°C	Overnight
Removal of cover glass	—	—
Nuclear counterstaining with DAPI	4′,6‐diamidino‐2‐phenylindole (DAPI) as per the manufacturer's instructions	—

The sealed samples were imaged with an AxioImager Z1 fluorescence microscope (Zeiss, Oberkochen, Germany) with filters specific for DAPI fluorescence (excitation 360 ± 20 nm, emission 460–25 nm), SpectrumGreen (excitation 480 ± 15 nm, emission 535 ± 20 nm), SpectrumOrange (excitation 538 ± 10 nm, emission 575 ± 15 nm). Hybridization signals were quantified in 25 non‐overlapping cell nuclei per specimen in each tissue punch, and count values were averaged for each triplicate. Brightfield microscopy was used, if required, to verify the presence of malignant or benign oral tissue in the field of view. The hybridization signals of 50 non‐overlapping nuclei were manually counted on a single‐cell basis. The results are presented as PD‐L1 gene signals per cell and calculated as the FISH ratio (PD‐L1 gene signals/chromosome 9 signals).

### 2.4 Statistical Analysis

Statistical analysis was conducted using IBM SPSS Statistics version 29 (IBM Deutschland GmbH, Ehningen, Germany). Pearson's chi‐squared test or Fisher's exact test was used to evaluate associations between clinical data and immunohistochemical biomarkers. Univariate survival analyses were performed using the Kaplan–Meier method, and the log‐rank test was used to compare survival distributions. Survival analyses were conducted for overall survival (OS) and tumor‐specific survival (TSS). OS was defined as the time from diagnosis to death from any cause, while TSS was defined as the time from diagnosis to tumor‐related death. All reported *p* values are two‐sided, and we considered a *p* value of 0.05 or less as statistically significant.

## 3 Results

### 3.1 Patient Characteristics

The final cohort included 130 patients with OSCC of various localizations. The cohort comprised 83 men (63.8%) and 47 women (36.2%) who underwent surgery at the Department of Oral and Maxillofacial Surgery of the University Hospital of Regensburg. Adjuvant radiotherapy or chemoradiotherapy was administered to 63 patients (48.4%). The mean age at diagnosis was 62.63 years (range: 32.91–88.32 years), and the mean follow‐up time was 3.61 years (range: 0.17–13.34 years). Out of the total number of patients, 38.5% reported never smoking, while 46.9% reported never drinking. A total of 45 (34.6%) patients were categorized as NS and ND. The mean age of the NSND group was 66.08 years (ranging from 32.91 to 88.32 years). The mean age of the SD group was 60.69 years (ranging from 35.58 to 86.62 years).

Determination of p16, CD4, CD8, CD1a, and FoxP3 expression was previously performed [6]. Out of the total of 130 patients, 10 (7.7%) were found to be p16 positive. FoxP3 was strongly expressed in 50% of the tumors, while CD1a was strongly expressed in 47.7% of them. CD4 was highly expressed in 50% of the samples and CD8 was highly expressed in 66 (50.8%) of the samples.

### 3.2 Association of Immunohistochemical Expression of PD‐L1, PD‐1, and Smoking and Drinking Status

**FIGURE 1:**
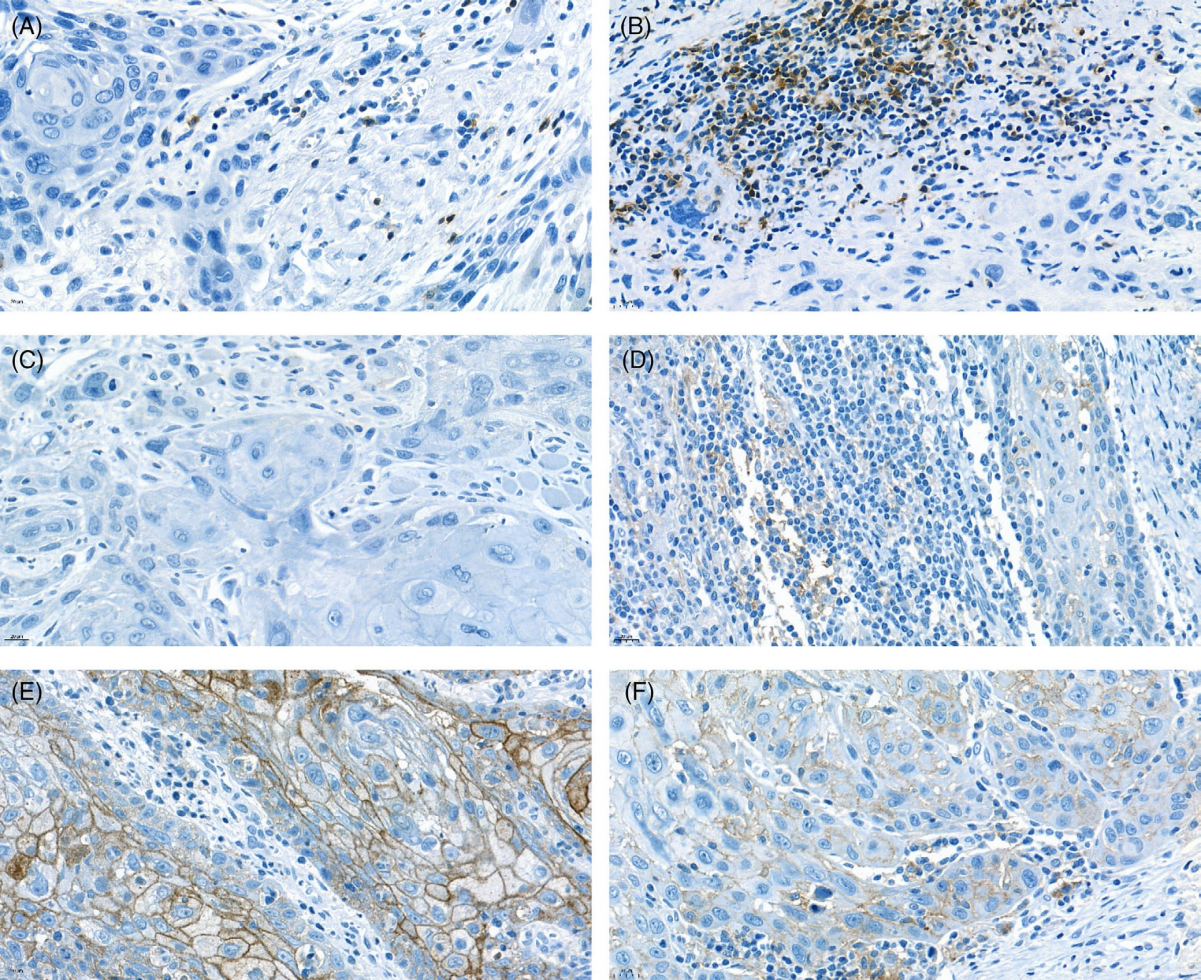
Examples of biopsy specimens from patients with oral squamous cell carcinomas. Examples with low PD‐1 expression (a), high PD‐1 expression (b), PD‐L1: TPS = 0%, ICS = 0% and CPS = 0 (c), PD‐L1: TPS < 1%, ICS = 10%–20%, CPS = 15% (d), PD‐L1: TPS = 50%, ICS < 1%, CPS = 95 (e), and TPS = 50%, ICS = 1%–5%, CPS = 70 (f), (magnification 400 ×). [Color figure can be viewed at wileyonlinelibrary.com]

PD‐1 and PD‐L1 expression evaluation was possible in all cases. High PD‐1 expression was detected in 64 cases (49.2%). PD‐L1‐TPS was positive in 16 cases (12.3%), while PD‐L1‐ICS was positive in 48 cases (36.9%). PD‐L1‐CPS was positive in 68 cases (52.3%) (Figure 1).

An overview of the results is shown in Table 2. The studies showed that high PD‐1 expression was significantly more frequent in NS (*p* = 0.011), ND (*p* < 0.001), as well as in the overall NSND group (*p* = 0.003). High PD‐L1‐TPS was significantly more frequent both in the overall NSND group (*p* = 0.044) and the ND group (*p* = 0.012). A significant association of PD‐L1‐ICS was only found for smoking, where high PD‐L1 expression was more frequent in NS (*p* = 0.042). Although the ICS was also increased in the NSND group, it did not reach significance (*p* = 0.054). A high PD‐L1‐CPS was significantly more common in both the NS and ND groups (*p* < 0.001 for each). The overall analysis of the NSND group also showed a highly significant association with a high CPS (*p* < 0.001).

**TABLE 2:** Relationship between smoking and drinking status and immunohistochemical PD‐L1 and PD‐1 expression.

	Tobacco				Alcohol				NSND			
	Never‐smoker	Smoker	*N*	*p*	Never drinker	Drinker	*N*	*p*	Yes	No	*N*	*p*
**PD‐1**												
Low	18 (36.0%)	48 (60.0%)	66 (50.8%)		21 (34.4%)	45 (67.2%)	66 (51.6%)		15 (33.3%)	51 (61.4%)	66 (51.6%)	
High	32 (64.0%)	32 (40.0%)	64 (49.2%)	**0.011**	40 (65.6%)	22 (32.8%)	62 (48.4%)	**< 0.001**	30 (66.7%)	32 (38.6%)	62 (48.4%)	**0.003**
**PD‐L1 TPS**												
Low	41 (82.0%)	73 (91.3%)	114 (87.7%)		49 (80.3%)	64 (95.5%)	113 (88.3%)		36 (80.0%)	77 (92.8%)	113 (88.3%)	
High	9 (18.0%)	7 (8.8%)	16 (12.3%)	0.169	12 (19.7%)	3 (4.5%)	15 (11.7%)	**0.012**	9 (20.0%)	6 (7.2%)	15 (11.7%)	**0.044**
**PD‐L1 ICS**												
Low	26 (52.0%)	56 (70.0%)	82 (63.1%)		35 (57.4%)	46 (68,7%)	81 (63.3%)		23 (51.1%)	58 (69.9%)	81 (63.3%)	
High	24 (48.0%)	24 (30.0%)	48 (36.9%)	**0.042**	26 (42.6%)	21 (31,3%)	47 (36.7%)	0.203	22 (48.9%)	25 (30.1%)	47 (36.7%)	0.054
**PD‐L1 CPS**												
Low	12 (24.0%)	50 (62.5%)	62 (47.7%)		16 (26.2%)	45 (67.2%)	61 (47.7%)		9 (20.0%)	52 (62.7%)	61 (47.7%)	
High	38 (76.0%)	30 (37.5%)	68 (52.3%)	**< 0.001**	45 (73.8%)	22 (32.8%)	67 (52.3%)	**< 0.001**	36 (80.0%)	31 (37.3%)	67 (52.3%)	**< 0.001**

*Note*: Significant values are presented in bold.

### 3.3 Association of Immunohistochemical Expression of PD‐L1, PD‐1 and Clinicohistopathologic Characteristics

The analysis of associations between PD‐L1 and PD‐1 expression with clinical parameters showed a significant association between low PD‐1 expression and advanced T‐stages (T3 + T4) (*p* = 0.032), as well as the presence of lymphangiosis carcinomatosa (*p* = 0.008). High PD‐1 expression was found to be associated with the presence of OPMD before tumor diagnosis (*p* = 0.004). High PD‐L1‐CPS was more prevalent in women (*p* = 0.010) and in older patients (≥ 71 years) (*p* = 0.002). Tumors located in the maxillary and mandibular alveolar ridge and the vestibule had higher prevalence of both high ICS (*p* = 0.001) and high CPS (*p* = 0.002). High CPS was also significantly more common in the tongue and soft palate.

### 3.4 Association of Immunohistochemical Expression of PD‐L1 and PD‐1 With TILs and p16

High PD‐1 expression was significantly more frequent in patients with high CD4, CD8, and total T‐cell expression (CD4 + CD8) (each *p* < 0.001). Additionally, associations with high CD1a expression (*p* = 0.035) and high FoxP3 expression (*p* < 0.001) were observed. High CD4 expression was significantly associated with high PD‐L1‐TPS (*p* = 0.014), high ICS (*p* < 0.001), and high CPS (*p* < 0.001). Similar results were found for the relationship between PD‐L1 expression and CD8 expression. High PD‐L1‐TPS, ICS, and CPS were each associated with high CD8 expression (*p* < 0.001 in each case). Total T‐cell expression also correlated significantly with PD‐L1 expression. High T‐cell expressions were associated with high ICS (*p* < 0.001), TPS (*p* = 0.001), and CPS (*p* < 0.001). No significant associations were found between PD‐L1 and CD1a expression. However, high PD‐L1‐ICS and CPS were significantly more frequent in patients with high FoxP3 expression (*p* < 0.001 each). No associations between PD‐1 or PD‐L1 Expression and p16 were found.

### 3.5 Properties of PD‐L1 Gene Copy Number

To evaluate the scattering range in normal tissue and determine the threshold for pathological gene amplification, we examined the copy numbers of the *PD‐L1* and *cen9* genes in 22 samples of healthy mucosa from the head and neck region. The *PD‐L1* gene copy number in healthy tissue ranged from 1.20 to 2.08 (mean = 1.58, SD = 0.18). The *cen9* hybridization values had a mean value of 1.47 (range: 1.24–1.71, SD = 0.16). In healthy tissue, the mean value of the ratio of *PD‐L1* and *cen9* was 1.08 (range: 0.90–1.21) with a SD of 0.10. These thresholds were used to define changes in *PD‐L1* gene copy number in OSCC. An increased *PD‐L1* gene copy number was defined as a value > 1.94, which corresponds to the mean value in healthy tissue plus two times the SD. A *PD‐L1* gene loss was defined as a value less than 1.22, which corresponds to the mean value in healthy tissue minus two times the SD.

**FIGURE 2:**
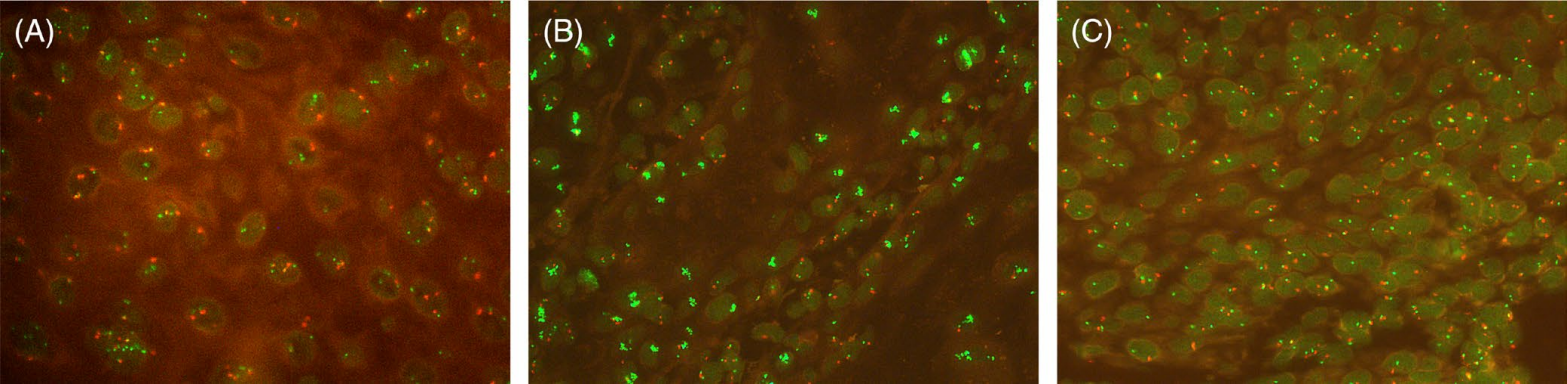
Fluorescence in situ‐hybridization (FISH) for PD‐L1. *PD‐L1* gene is labeled in green, centromer 9 in red. FISH analysis showing *PD‐L1* polysomy (a), *PD‐L1* amplification (b), and *PD‐L1* disomy (c), (magnification 400 ×). [Color figure can be viewed at wileyonlinelibrary.com]

In this study, 40 patients (30.8%) had an increased *PD‐L1* gene copy number, while five patients (3.8%) had a loss of *PD‐L1*. Only eight patients (6.2%) had actual *PD‐L1* amplification, defined as a *PD‐L1/cen9* ratio > 2 (Figure 2). Polysomy was defined as more than two *PD‐L1* gene copies with a *PD‐L1/cen9* ratio < 2, which was found in 20.8% of patients.

### 3.6 Associations of PD‐L1 Gene Copy Number and Smoking and Drinking Status

*PD‐L1* was increased in both NS and ND, but the trend did not reach significance (*p* = 0.081 for ND and *p* = 0.054 for NS). There was a similar non‐significant association of increased *PD‐L1* gene copy number in the overall NSND group (*p* = 0.108). No significant correlations were found between *PD‐L1* loss and smoking or drinking. However, all patients with *PD‐L1* loss were represented in the ND (*p* = 0.054) and NS (*p* = 0.154) groups.

### 3.7 Associations of PD‐L1 Gene Copy Number and Clinicohistopathologic Characteristics

Increased *PD‐L1* gene copy number and the presence of *PD‐L1* polysomy were associated with lymphangiosis carcinomatosa (both *p* = 0.048). The study also revealed a higher incidence of recurrences in patients with an increased *PD‐L1* gene copy number (*p* = 0.024).

### 3.8 Associations of PD‐L1 Gene Copy Number and Immunohistochemical Expression of PD‐L1 and PD‐1, TILs and p16

Significant correlations were identified between the PD‐L1 gene and immunohistochemical PD‐L1 protein expression (Table 3). Patients with increased *PD‐L1* gene copy number and especially those with amplification had a positive PD‐L1‐TPS (*p* = 0.039 and *p* < 0.001). *PD‐L1* gene copy loss was in turn associated with a negative PD‐L1‐CPS (*p* = 0.023).

**TABLE 3:** Correlation between *PD‐L1* gene amplification, polysomy and *PD‐L1* gene copy number with PD‐L1 protein expression.

	PD‐L1 gene copy gain				PD‐L1 gene copy loss				PD‐L1 amplification				PD‐L1 polysomy			
	No	Yes	*N*	*p*	No	Yes	*N*	*p*	No	Yes	*N*	*p*	No	Yes	*N*	*p*
**PD‐L1 TPS**																
Low	83 (92.2%)	31 (77.5%)	114 (87.7%)		109 (87.2%)	5 (100.0%)	114 (87,7%)		111 (91.0%)	3 (37.5%)	114 (87.7%)		91 (88.3%)	23 (85.2%)	114 (87.7%)	
High	7 (7.8%)	9 (22.5%)	16 (12.3%)	**0.039**	16 (12.8%)	0 (0.0%)	16 (12.3%)	1000	11 (9.0%)	5 (62.5%)	16 (12.3%)	**< 0.001**	12 (11.7%)	4 (14.8%)	16 (12.3%)	0.742
**PD‐L1 ICS**																
Low	54 (60.0%)	28 (70.0%)	82 (63.1%)		77 (61.6%)	5 (100.0%)	82 (63.1%)		77 (63.1%)	5 (62.5%)	82 (63.1%)		63 (61.2%)	19 (70.4%)	82 (63.1%)	
High	36 (40.0)	12 (30.0)	48 (36.9%)	0.328	48 (38.4%)	0 (0.0%)	48 (36.9%)	0.157	45 (36.9%)	3 (37.5%)	48 (36.9%)	1000	40 (38.8%)	8 (29.6%)	48 (36.9%)	0.502
**PD‐L1 CPS**																
Low	44 (48.9%)	18 (45.0%)	62 (47.7%)		57 (45.6%)	5 (100.0%)	62 (47.7%)		60 (49.2%)	2 (25.0%)	62 (47.7%)		50 (48.5%)	12 (44.4%)	62 (47.7%)	
High	46 (51.1%)	22 (55.0%)	68 (52.3%)	0.708	68 (54.4%)	0 (0.0%)	68 (52.3%)	**0.023**	62 (50.8%)	6 (75.0%)	68 (52.3%)	0.278	53 (51.5%)	15 (55.6%)	68 (52.3%)	0.829

*Note*: Significant values are presented in bold.

In addition, significant associations were found between *PD‐L1* gene copy number and the other immunohistochemical markers examined. Specifically, *PD‐L1* loss of tumor cells was significantly associated with lower tumor infiltration of CD8+ T‐cells (*p* = 0.027). Additionally, patients with *PD‐L1* amplification showed reduced tumor infiltration with CD1a+ cells (*p* = 0.007).

### 3.9 Survival Analysis

**FIGURE 3:**
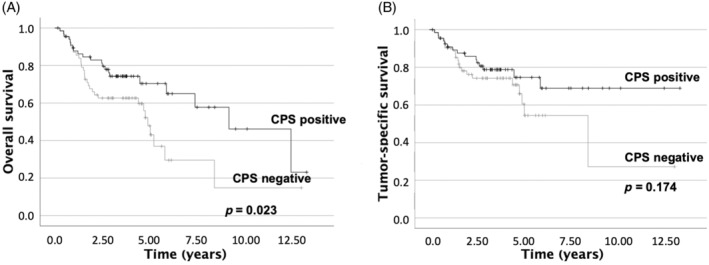
Univariate survival analyses. High PD‐L1 CPS is associated with better overall survival **(**a) and tumor‐specific survival (b).

Univariate survival analyses were conducted for overall survival and tumor‐specific survival for both immunohistochemical PD‐L1 expression and FISH *PD‐L1* expression. The results revealed that positive PD‐L1‐CPS was significantly associated with better overall survival (*p* = 0.023) (Figure 3).

## 4 Discussion

The introduction of PD‐L1/PD‐1 checkpoint therapies has ushered in a new era in cancer treatment. However, not all patients show the same results when treated [14, 15]. Therefore, current research aims to identify patients who will benefit most from checkpoint therapy to significantly improve outcomes. Previous studies have shown that NSND‐OSCCs have different clinical and immunologic characteristics compared to SD associated OSCCs [5, 6, 19]. Among others the NSND‐OSCC has higher numbers of TILs, including CD4‐positive, CD8‐positive, and FoxP3‐positive‐cells, which indicates that the NSND‐OSCC is a highly immunological active tumor [6]. Thus, the use of immunotherapies may be particularly promising in this patient population. Pembrolizumab and nivolumab are anti‐PD‐1 checkpoint inhibitors that have been established for use in first‐ and second‐line palliative therapy regimens in OSCC [15]. Currently, there is limited data on the relationship between the efficacy of immune‐checkpoint inhibitors and a patient's history of smoking and alcohol use. However, a Phase III trial of nivolumab in patients with recurrent HNSCC suggests that the survival benefit associated with nivolumab is numerically lower in smokers compared to NS [20]. PD‐1 is normally expressed on T‐cells, B‐cells and myeloid cells [21, 22]. In this study high PD‐1 expression was associated with NS and ND. This correlation could be due to the overall higher number of TILs in the NSND‐OSCC group [6]. Consequently, blocking the PD‐1/PD‐L1‐checkpoint may induce a particularly strong immune response. Therapy decisions for checkpoint inhibitors are currently based on clinical studies that determined PD‐L1 expression through immunohistochemistry. Checkpoint therapy is chosen if the CPS is ≥ 1 or the TPS is ≥ 50% [15, 23, 24, 25]. In the present study we assessed PD‐L1 expression on tumor cells (TPS), immune cells (ICS), and the combination (CPS). To maximize the clinical relevance, we chose the same thresholds of ≥ 1 (CPS) and ≥ 50% (TPS) as are in clinical use. Only a minority of patients had a positive TPS at 12.3%. However, the NSND‐OSCC was significantly more common in this group. A positive PD‐L1‐CPS was identified in approximately half (52.3%) of the patients. Once again, NSND patients were significantly more likely to have positive PD‐L1 levels. Additionally, the PD‐L1‐ICS, that represents the PD‐L1 expression of immune cells, was also associated with NSND. Our findings are in line with previous research, which has demonstrated that the NSND groups exhibit elevated PD‐1 signaling and increased PD‐L1 expression [5, 19, 26, 27]. One possible reason for the increased PD‐L1 expression in NSND could be an upregulation due to increased interferon‐γ a signaling, which was also found to be associated with NS in previous studies [5, 19]. In this case, PD‐L1 expression is dynamic and exhibits variable, time‐dependent changes [28]. On the other hand, gene amplification events involving the 9p24.1 locus, which contains the *CD274/PD‐L1* gene, can drive PD‐L1 expression [28]. In the present study we demonstrated that NSND patients had a higher incidence of increased *PD‐L1* gene copy number along with the increased PD‐L1 protein expression. Additionally, *PD‐L1* loss was only observed in SD patients. Although significance was not achieved in this study, these findings suggest a potential influence of smoking and drinking on *PD‐L1* gene copy number changes in OSCC. Interestingly, the relationship between smoking and the immunologic microenvironment, as well as PD‐L1 expression, appears to depend on the tumor entity. For example, in lung cancer, the correlation between PD‐L1 expression and smoking status is inverse to our findings in OSCC [29]. Smoking causes DNA damage, resulting in a higher tumor mutational burden [26]. In HNSCC a higher mutational signature of smoking was associated with a lower level of cytolytic activity of immune infiltration and interferon‐*γ* signaling [26]. However, in SCC of the lung, these relationships were reversed [26].

The current study also showed correlations of PD‐1 and PD‐L1 with clinical parameters and patient outcomes, which have previously been observed more frequently in the NSND‐OSCC group [6]. Tumors with high PD‐1 expression were often preceded by OPMD and occurred more frequently in women and older patients. In addition, high PD‐L1 expression was associated with better survival and appears to be dependent on the tumor location in the oral cavity. High expression was found in the upper and lower jaw, the tongue and the soft palate. Locations which also occur more frequently in NSND‐OSCC [6].

Currently, there is limited information regarding the significance of *PD‐L1* gene copy number in OSCC. We found an enhanced *PD‐L1* gene copy number in approximately one third of the patients. In most cases, a polysomy of *PD‐L1* was found, while only eight patients showed actual *PD‐L1* amplification. Additionally, five of the examined tumors showed a *PD‐L1* loss. Interestingly, we demonstrated that the PD‐L1 gene copy number was associated with PD‐L1 protein expression. Specifically, an amplification of *PD‐L1* appears to upregulate the protein expression. A study conducted by Straub et al. showed similar results [28]. However, like the current study, there were exceptions, and not all tumors with gene amplification showed high PD‐L1 protein expression [28]. Post‐transcriptional splicing and methylation are potential mechanisms that limit the protein expression [30]. The presence of gene amplification with no or low‐level PD‐L1 protein expression may reduce the effectiveness of immune‐checkpoint inhibitors [30]. However, it has been demonstrated in other tumor entities that tumors with *PD‐L1* amplification and lack of PD‐L1 protein expression can also benefit from immune checkpoint therapy [30]. Technical problems in the immunohistochemical analysis, such as tumor heterogeneity or differences in the affinity of various anti‐PD‐L1 antibodies, could be possible reasons [30]. These may impact the accuracy of protein expression methods and clarify why some patients do not exhibit PD‐L1 expression in immunohistochemical analysis. Additionally, other mechanisms, such as the expression of PD‐L2 instead of PD‐L1, may also contribute to patient response to anti‐PD‐1 agents despite lacking PD‐L1 expression [30]. In the present study we also showed that loss of *PD‐L1* can lead to a decrease in PD‐L1 protein expression on both tumor and immune cells. The 9p24.1 locus, which also contains *PD‐L1*, has been shown to contribute significantly to an immunocold or immunohot phenotype (when genes are lost or gained, respectively) in HPV‐negative HNSCC [31]. The current results are consistent with this finding: the loss of *PD‐L1* was linked to a lower number of tumor‐infiltrating CD8‐positive‐cells, while *PD‐L1* amplification was associated with a higher number of tumor‐infiltrating dendritic cells. Additional studies are required to examine the role of *PD‐L1 gene* copy number as a predictor of response to immune‐checkpoint therapy.

In summary, patients with NSND‐OSCC may benefit from immune checkpoint therapy due to their immunological status. Additionally, surveying the *PD‐L1* gene copy number before planned checkpoint therapy could help identify patients who may benefit from immune‐checkpoint therapy despite a lack of protein expression. These conclusions require further investigations in future studies.

## Author Contributions

Study concept: Mathias Fiedler and Tobias Ettl. Study design: Mathias Fiedler and Tobias Ettl. Data acquisition: Mathias Fiedler, Alisa Off, Gärtner Andreas, Gero Brockhoff, Richard Bauer, and Weber Florian. Quality control of data: Mathias Fiedler, Richard Bauer, Tobias Ettl, Florian Weber, Jonas Eichberger, Johannes Schuderer, Michael Maurer, and Maximillian Gottsauner. Data analysis and interpretation: Mathias Fiedler, Tobias Ettl, and Weber Florian. Manuscript preparation: Mathias Fiedler. Manuscript editing: Mathias Fiedler and Florian Weber. Manuscript review: All authors.

## Ethics Statement

Approval of the ethics committee of the University of Regensburg: No. 22–2798‐101.

## Consent

The authors have nothing to report.

## Conflicts of Interest

The authors declare no conflicts of interest.

## Data Availability

The data that support the findings of this study are available from the corresponding author upon reasonable request.
